# Spatial Patterns of *Adelges tsugae* Annand (Hemiptera: Adelgidae) in Eastern Hemlock Stands: Implications for Sampling and Management

**DOI:** 10.3390/insects15100751

**Published:** 2024-09-28

**Authors:** Sunghoon Baek, Yong-Lak Park

**Affiliations:** 1 Entomology Program, School of Natural Resources & the Environment, West Virginia University, Morgantown, WV 26505, USA; shbaek007@hotmail.com; 2 Agro-Environment Research Institute, Epinet Co., Ltd., Anyang-si 14054, Republic of Korea

**Keywords:** *Adelges tsugae*, sampling unit, coefficient of variation, spatial distribution, geostatistics, SADIE, semivariogram

## Abstract

**Simple Summary:**

Understanding where insect pests inhabit and how they interact with their environment is important for creating sampling and management plans. This study focused on the hemlock woolly adelgid, a pest that affects hemlock trees, to find the best way to sample these pests and understand their distribution in hemlock stands in the USA. We randomly selected 24 branches from each of 46 infested hemlock trees and counted the egg sacs of hemlock woolly adelgid on each branch to determine the best sampling unit. We also divided three hemlock forest areas into grids and counted the egg sacs in each grid cell to study the spatial distribution pattern of hemlock woolly adelgid. This study found that hemlock woolly adelgids tended to aggregate at the tips of branches and that a 50 cm branch from the lower canopy was the best sampling unit. The hemlock woolly adelgid also showed spatial aggregation in the hemlock stands, and its abundance was correlated with tree height and tree diameter at breast height. These findings help in better sampling and managing hemlock woolly adelgid.

**Abstract:**

Understanding the spatial patterns of insect pests and their associations with their environments is crucial for developing effective sampling and management plans. This study was conducted to identify optimal sampling units for the hemlock woolly adelgid, *Adelges tsugae* Annand (Hemiptera: Adelgidae) and to characterize its spatial distribution patterns in hemlock (*Tsuga canadensis* (L.) Carrière) stands in West Virginia, USA. To determine the optimal sampling unit, we randomly selected 24 branches from each of 46 *A. tsugae*-infested hemlock trees. The locations and number of *A. tsugae* ovisacs on each branch were recorded and the coefficient of variation was used to choose the optimal sampling units. To determine the spatial patterns of *A. tsugae*, each of the three 1-ha hemlock stands was divided into 100 grids, and ovisac counts were taken from the center of each grid. Semivariograms and spatial analysis by distance indices (SADIE) were used to analyze the spatial patterns of *A. tsugae*. In addition, various environmental and biological factors were measured to explore their spatial associations with *A. tsugae*. The results of this study revealed that the *A. tsugae* ovisacs exhibited spatial aggregation within branches, predominantly at the tips, and a 50 cm branch approximately 3 m above the ground was the optimal sampling unit. The spatial aggregation of *A. tsugae* in the hemlock stands was evident, and positive spatial associations were found between *A. tsugae* populations and factors including the aspect, tree diameter at breast height, and tree height. These findings offer valuable insights for the sampling and management of *A. tsugae*.

## 1. Introduction

The hemlock woolly adelgid, *Adelges tsugae* Annand (Hemiptera: Adelgidae), is a major pest of *Tsuga canadensis* ((L.) Carrière (Eastern hemlock)) in the eastern USA [1,2]. *T. canadensis* is shade-tolerant and a dominant plant species in the forest system of North America [1,3]. In a hemlock-dominated forest, only 5% of sunlight can reach the understory [4], and soils exhibit low pH, high C:N ratios, and a low rate of N mineralization and nitrification [5,6]. These conditions create unique microclimates which provide favorable habitats for certain animals and affect understory plant species’ composition, biomass, and productivity among various hemlock stands [3]. Therefore, the continuous loss of hemlock can cause a change in the ecosystem properties of hemlock-dominant forests. These important hemlock-dominant forests have been threatened by *A. tsugae*. Heavy infestations of *A. tsugae* cause poor crown health and reduced shoot growth that, in combination with other environmental stresses, can result in rapid tree decline and death [7,8]. Almost 90% of the geographic range of eastern hemlock has been infested with *A. tsugae* in North America [8,9]. Further, the limited effects of *A. tsugae*’s natural enemies, parthenogenetic reproduction of *A. tsugae*, and susceptibility of *T. canadensis* to *A. tsugae* allow *A. tsugae* populations to build up quickly [10,11]. Currently, *A. tsugae* management focuses mainly on treating individual trees with insecticide and biological control agents including predatory insects and entomopathogenic fungi [12].

*Adelges tsugae* annually produces two parthenogenetic generations on hemlock. The progrediens generation occurs in spring and the sistens generation persists from summer to the following spring. In June and July, the sistens nymphs emerge as crawlers from eggs and settle at the base of young needles where they immediately enter aestivation (i.e., summer diapause) [7,13]. They resume development in October and complete their development in late winter. The adults of the sistens generation produce eggs from March until May and their progenies become winged or wingless adults depending on the density of *A. tsugae* [14]. The winged adults disperse to tigertail spruce (*Picea polita* (Siebold & Zucc.) Carrière), but no suitable alternate hosts are known in the eastern USA. Therefore, winged sexuparae are known to die without reproduction in the USA [14]. The wingless progrediens remain on hemlock, complete their development, and lay sistens eggs in June [14].

One of the most important steps in integrated pest management (IPM) is sampling to estimate the population density of a target pest, delimit its distribution, and determine where to apply control measures [15]. Because counting individual *A. tsugae* or its ovisacs on an entire tree is not feasible, sampling from an *A. tsugae* population is commonly used to estimate its density. Depending on the size and nature of the sampling unit, the sampling techniques used, the number of samples required, the spatial pattern of the sample layout, and the sampling timing can be determined [15]. In general, a sampling unit is determined based on multiple criteria (e.g., consistency, suitable size, stability, and the possibility of delineation in the field), including a balance between cost and precision [16,17,18].

A few previous studies [1,19,20,21] investigated the within-tree distribution of *A. tsugae*, which is critical information that directly affects the selection of sampling units and the application of control measures. Fidgen et al. [1] hypothesized that the density of *A. tsugae* in the middle crown of hemlock would not differ from that of the lower crown. However, Evans and Gregoire [17] showed that the density of *A. tsugae* was slightly higher on the lower branches than on the upper branches when *A. tsugae* density was high, while a higher density was found on the upper branches with a low level of *A. tsugae* infestation. Whitmore [20] concluded that more *A. tsugae* were found on lower branches based on his hypothesis that the crawlers of *A. tsugae* became established at any place within a tree crown but that *A. tsugae* would usually be found on the lower branches as the crawlers fell downward through the canopy after a few generations. Joseph et al. [21] reported that *A. tsugae* ovisacs were more abundant on the higher branches in insecticide-untreated trees, but their within-tree distribution changed over time. These previous studies [1,19,20,21] showed an inconsistent *A. tsugae* distribution within a hemlock tree.

Furthermore, a few other studies described the within-tree distribution and spatial dispersion of *A. tsugae* in hemlock stands. Fidgen et al. [22] reported the spatial aggregation of *A. tsugae* using Taylor’s power law, aiming at the development of sampling plans. Although understanding spatial aggregation is useful for developing effective sampling programs by allowing estimation of the sampling accuracy, efficiency, and precision, using distribution indices such as Taylor’s power law does not consider the spatial locations of samples. So far, the spatial distribution of *A. tsugae* has not been characterized using geostatistical approaches that use information on the spatial location of each sample and which can thus describe and visualize the spatial pattern of *A. tsugae*. In addition, understanding the spatial distribution of *A. tsugae* and its relationship with environmental factors could be the key to efficiently and effectively managing *A. tsugae*. A few previous studies attempted to predict *A. tsugae* damage based on some environmental factors. Evans and Gregoire [23] considered the plant hardiness zone, elevation, forest cover type, urbanization, precipitation, temperature, and geographic range of eastern hemlock to predict the spread of *A. tsugae.* Other studies [24,25,26] found that roads, major trails, riparian corridors, latitudes, and aspects were significantly related to the abundance of *A. tsugae.* Although these studies are useful for detecting and predicting the infestation of *A. tsugae,* the direct spatial association between *A. tsugae* abundance and environmental factors has not been fully explained.

To address the current sampling issues mentioned above, we conducted three-year studies to determine the optimal sampling unit for *A. tsugae* ovisacs by investigating their within-branch and within-tree distributions (study 1) and to characterize and describe the spatial distribution patterns of *A. tsugae* (study 2). The within-tree distribution of *A. tsugae* would help to determine which branches within a hemlock tree need to be sampled, and the within-branch distribution of *A. tsugae* would suggest what size and part of a branch need to be sampled (study 1). To help park managers and foresters prioritize areas for management, we characterized spatial distribution patterns of *A. tsugae* ovisacs and determined the spatial association between *A. tsugae* ovisacs and environmental factors (study 2). Because *A. tsugae* management with pesticides and biological control agents is expensive and time-consuming, prioritizing areas for the application of control measures using the spatial distribution of *A. tsugae* would increase the efficiency and effectiveness of *A. tsugae* management (i.e., spatially targeted or site-specific management).

## 2. Materials and Methods

### 2.1. Study 1: Determining Optimal Sampling Unit for A. tsugae Ovisac Sampling

#### 2.1.1. Study Sites

This study was conducted in Blackwater Falls State Park (N39°6′25′′, W79°30’13′′) and Cathedral State Park (N39°19’44′′, W79°32′24′′) in West Virginia, USA, and Buchannan State Forest (N39°57′32′′, W77°57′0′′) in Pennsylvania, USA. No *A. tsugae* management was done in the three sites during the study period.

#### 2.1.2. Sampling

Sampling *A. tsugae* ovisacs was conducted in two ways: sampling 100 cm branches and whole branches. The 100 cm branch is commonly used as a sampling unit for *A. tsugae* detection [27]. A total of 24 branches per tree were randomly selected at six different vertical heights (i.e., two levels of height for each of upper, middle, and lower crowns) and four cardinal directions (i.e., east, west, south, and north). Before cutting the selected branches with a 10 m pole pruner, the tree height and the height of each selected branch within the tree were measured with a clinometer (EC II D, Haglöf; Långsele, Sweden) by measuring the angle of the top and bottom of each tree from a fixed distance (e.g., 10 m for small tree and 25 m for tall tree) from the target tree. All samples were transported to a cold chamber maintained at ca. 4 °C in the Entomology Laboratory at West Virginia University.

For 100 cm branch sampling, 31 *A. tsugae*-infested trees (3.5–7.0 m in height) were randomly selected from hemlock stands in the three study sites. A total of 504 branches in 21 trees were sampled on 7 April 2011 in Blackwater Falls State Park. These samples represent ovisacs of a progrediens generation of *A. tsugae.* Ovisacs of a sistens generation were sampled by collecting 120 branches from five trees in Cathedral State Park on 29 June 2011. A total of five tall (15.5–18.3 m) trees were sampled by felling trees in Buchanan State Forest on 1 June 2012.

For whole-branch sampling, 360 branches from 15 trees were collected at the three study sites. Tree height was 3.5–7.0 m in Cathedral and Blackwater Falls State Parks and 15.5–18 m in Buchanan State Forest. The sampling dates were 29 March 2013 in Cathedral State Park, 11 June 2013 in Buchanan State Forest, and 18 June 2013 in Blackwater Falls State Park.

#### 2.1.3. Characterizing the Within-Tree Distribution of *A. tsugae*

The effect of branch height and cardinal direction on the density of *A. tsugae* ovisacs was investigated with ANOVA followed by Tukey’s HSD test [28]. Two types of *A. tsugae* ovisac densities were used for the analysis: total ovisac numbers per branch and the number of ovisacs per 10 cm twig with foliage. The number of ovisacs per 10 cm twig with foliage was calculated by dividing the total number of ovisacs on a branch by the total length of twigs on the same branch. Because the data were not normally distributed, all data were square-root transformed before statistical analysis. Separate analyses were conducted for 10 cm-branch and whole-branch samples.

#### 2.1.4. Characterizing the Within-Branch Distribution of *A. tsugae*

**Figure 1:**
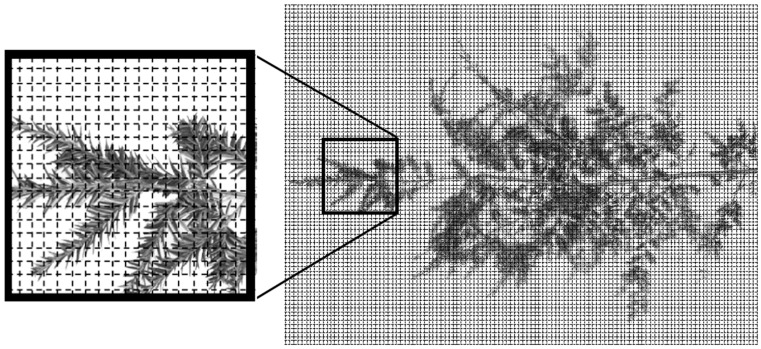
Method for assigning coordinates to the locations of *A. tsugae* ovisacs within a branch. Each grid is 1 cm × 1 cm and the number of *A. tsugae* ovisacs was counted to conduct spatial analysis and to map the within-branch distribution of *A. tsugae* ovisacs.

To determine the spatial distribution patterns of *A. tsugae* ovisacs within a branch, each 100 cm branch was assigned spatial coordinates using 1 cm by 1 cm grids (Figure 1) to assign spatial coordinates to ovisacs in the branch; hatched ovisacs were also included in the count. The branch length in each grid was measured to estimate habitable space for *A. tsugae*. Because the number of ovisacs was not statistically (*p* > 0.05) different among the four cardinal directions in each study site, data from each site were pooled for analyses. The distribution of *A. tsugae* ovisacs within branches was visualized with ordinary kriging, an interpolation method, in Geostatistics, in ArcGIS 9.3 (Environmental Systems Research Institute, Redlands, CA).

Spatial analysis by distance indices (SADIE) [29] was used to test the statistical significance of spatial aggregation, randomness, and uniformity of *A. tsugae* ovisacs within the branch. SADIE measures the degree of clustering with patches and gaps. A patch is a region with a relatively large number of ovisacs, and a gap is a region with a relatively small number of ovisacs. To quantify the degree of clustering of ovisacs within a branch, the overall aggregation index (*I*_a_) was calculated as described in Perry et al. [30]: *I*_a_ = 1 for random pattern, *I*_a_ > 1 for aggregated pattern, and *I*_a_ < 1 for uniform pattern. The significance test for spatial patterns was conducted with formal randomization tests [30,31] under the null hypothesis that the observed ovisacs were arranged randomly among the given sample locations. SADIE also quantifies the contribution of each ovisac count at each tree to a patch or a gap with unitless clustering indices [30], where *v_i_* = 1 or │*v_j_*│= 1 for random distribution of ovisacs within a stand, *v_i_* > 1 for belonging to a patch, and *v_j_* < −1 for belonging to a gap. This study used two thresholds, 1.5 for vi and −1.5 for vj, to map patches and gaps of *A. tsugae* ovisacs within a stand [30]. All SADIE statistics were calculated with SADIEShell version 1.22 (Rothamsted Experimental Station, Harpenden, Herts, UK). ArcGIS version 9.3 (ESRI, Redlands, CA, USA) was used to map the patches and gaps.

#### 2.1.5. Determining Sampling Units for *A. tsugae* Ovisacs

To determine the optimal sampling unit, the coefficients of variation (CVs) of *A. tsugae* ovisacs were compared among six branch heights and three branch lengths (i.e., 25, 50, and 100 cm). CV is the ratio of the standard deviation to the mean, and a low CV value indicates that the estimated density is more precise compared to that of other sampling units acquired with similar means. In this study, the effect of the cardinal direction was not considered for CV calculation because there was no significant (*p* > 0.05) difference in the number of *A. tsugae* ovisacs among the directions. All statistical analyses were conducted separately for each site and two branch samplings (i.e., whole branch and 100 cm branch) because the total number per branch (*F* = 4.85; df = 2, 43; *p* = 0.02) and number of *A. tsugae* ovisacs per 10 cm twig with foliage (*F* = 4.25; df = 2, 43; *p* = 0.02) were statistically different among the sites.

#### 2.1.6. Estimating Within-Branch Abundance of *A. tsugae* Ovisacs

To estimate the total number of *A. tsugae* ovisacs within a branch, the cumulative ovisac numbers at various distances from the tip of a branch were modeled using the data from whole-branch samples. The modeling was done by using the Weibull function [32], described as *f* (*x*) = 100 [1 − exp (*−* [*x − a*]*^b^*)], where *f* (*x*) is the cumulative percentage of ovisac occurrence, *x* is branch length from the tip, and *a* and *b* are model parameters. Frequency data from ovisac counts for every 25 cm branch, from the tip of a branch, were converted to cumulative proportions and the parameters of the Weibull function were estimated with non-linear regression analysis [28].

### 2.2. Study 2: Determining Spatial Distribution Patterns of A. tsugae Ovisac

#### 2.2.1. Study Sites and Sampling *A. tsugae* and Environmental Factors

This study was conducted in the three sites used for study 1, but not in the same location within each of the three study sites: Blackwater Falls State Park, Cathedral State Park, and Buchannan State Forest

A systematic-fixed sampling was used in this study. Each study area was divided into 100 grids (i.e., 10 m by 10 m per grid), and a tree in the middle of each grid was selected for sampling. Two 50 cm branches of the tree approximately 3 m above the ground were cut and transported to a cold room maintained at 4 °C. This sampling was conducted at the peak of the ovisac number for the two *A. tsugae* generations (i.e., progrediens and sistens) from the summer of 2012 to the spring of 2014. The sampling dates were 6 June 2012, 5 April and 25 June 2013, and 2 May 2014 in Cathedral State Park; 8 June 2012, 26 March and 11 June 2013, and 9 April 2014 in Buchanan State Forest; 13 June 2012, 17 April and 3 July 2013, and 16 April 2014 in Blackwater Falls State Park.

Biotic and abiotic environmental variables were sampled to investigate their spatial association with *A. tsugae* ovisacs. Elevation was measured using a differentially corrected global positioning system (DGPS, Mobile Mapper pro, Magellan, Santa Clara, CA, USA), and the aspect was measured with a compass (Silva Expedition 4, Silva, Sweden) at the selected tree of each grid. The diameter at breast height (DBH) was measured by using a tape measure, and the tree height was measured with a clinometer as described in Section 2.1.2. The basal area of each grid was estimated by measuring the DBH of all trees later than the sapling stage: basal area = 0.00007854 × DBH^2^. All trees and shrubs in each sampling grid were identified to determine diversity using Simpson’s diversity index [33].

#### 2.2.2. Characterizing Spatial Distribution Patterns of *A. tsugae*

Spatial distribution patterns of *A. tsugae* ovisacs were characterized using empirical semivariograms that measure the spatial dependence of each datum. Spatial dependence or spatial correlation means that two sample values close to one another tend to be more similar than two values farther apart [34]. Semivariogram functions (i.e., semivariance) can be defined as:

(1) $$\gamma (h)=\frac{1}{2n(h)}\sum {\left[Z(x_{i})-Z(x_{i+h})\right]}^{2}$$

where h is the lag distance or separation distance, *Z*(*x_i_*) is the measured sample value at the sample point *x_i_*, *Z*(*x_i+h_*) is the sample value at *x_i+h_*, and *n*(*h*) is the total number of sample pairs for any separation distance. A semivariogram for each data set was generated using GS+ version 9 (Gammadesign, Plainwell, MI, USA). If the number of sample pairs at a certain lag distance was less than 30, the semivariance for that lag distance was excluded to obtain a robust semivariogram [35]. The best semivariogram models were selected based on r^2^ values for fitting the semivariogram models [36].

The spatial structure of *A. tsugae* ovisac distribution was characterized by three semivariogram parameters (i.e., nugget, sill, and range). The range is the distance at which the lag distance is beyond where samples are spatially independent. The sill is the value of the semivariance at any distance greater than or equal to the range. The nugget is the value of semivariance when lag distance equals zero, i.e., y-intercept in semivariogram [37]. The nugget value indicates the existence of experimental errors, random effects, and microscale variation. When a spatial trend or spatial drift exists in the data, semivariances increase continuously without a range or sill, indicating non-stationarity. To meet the assumption of stationarity, spatial trends in the data sets were removed [38,39] with trend-surface analysis. Linear surface trends were obtained with regression analysis, and the goodness-of-fit of the trend surface was tested with ANOVA using SigmaPlot 11 (Systat Software Inc, San Jose, CA, USA). If the trend was significant (*p* < 0.05), the residuals were used in semivariogram modeling. The degree of spatial dependence was quantified by calculating the percent variability explained by spatial dependence, (sill − nugget)/sill’ 100 [40].

Although the semivariogram quantifies spatial dependence, it does not test the significance of spatial distribution patterns. Thus, we used SADIE to determine the spatial pattern of *A. tsugae* ovisacs as described in Section 2.1.4.

#### 2.2.3. Spatial Associations between Generations of *A. tsugae*

SADIE was used for spatial association analyses [30] that compared the cluster indices for individual sample locations of two data sets. Positive association was indicated by the coincidence of a patch for one data set with a patch for the other, or by the coincidence of two gaps. A negative association was indicated by a patch coinciding with a gap. The measure of spatial association was represented by a SADIE index, X, which is the mean of the local correlation coefficient between the clustering indices of the two sets: X > 0 for a positive spatial association, X = 0 for no spatial association, and X < 0 for negative spatial association [31]. Positive X values indicate the coincidence of a patch cluster for one set with a patch cluster for the other or the coincidence of two gaps, and negative X values indicate a patch coinciding with a gap [31]. The associated possibility (*P*) was also calculated based on randomization tests [31] as part of the spatial aggregation analysis [30]. The null hypothesis is that the spatial arrangement of the count data between two data sets is random [39]. In this study, X > 0 indicates positive spatial association, X = 0 indicates no spatial association, and X < 0 indicates negative spatial association.

Because successive sampling was done at the same locations for two years, spatial association analysis was used to measure the temporal associations between spatial distributions of *A. tsugae* ovisacs. Analyzing spatial and temporal changes measured the persistence of spatial distribution of *A. tsugae* populations between generations. In this case, X > 0 indicates the persistence of spatial distribution, X ≈ 0 indicates no spatial relationship between time intervals, and X < 0 indicates an opposite distribution.

#### 2.2.4. Spatial Association of A. tsugae Ovisacs with Environmental Factors

The measurement of spatial association, a SADIE index (X), was used as described above to measure the spatial association between *A. tsugae* ovisacs and geographic factors (i.e., elevation and aspect), tree measurements (i.e., DBH and height), and environmental factors (i.e., basal area and diversity of each grid).

## 3. Results

### 3.1. Study 1: Determining Optimal Sampling Unit for A. tsugae Ovisac Sampling

#### 3.1.1. Within-Tree Distribution of *A. tsugae* Ovisacs

Regardless of whether we analyzed 100 cm or whole-branch samples, both the number and density of *A. tsugae* ovisacs were not significantly (*p* > 0.05) different among the four cardinal directions; however, significant (*p <* 0.05) differences were found among six heights in all three sites in most cases (Table 1). The upper branches had higher ovisac densities per branch and more habitable space than the middle and lower branches (Tables S1 and S2). We also found that the density of ovisacs increased from the lowest to highest branches for short trees (<8 m), but the density was the lowest in the middle branches for tall trees (>15 m).

#### 3.1.2. Within-Branch Distribution of *A. tsugae* Ovisac

**Figure 2:**
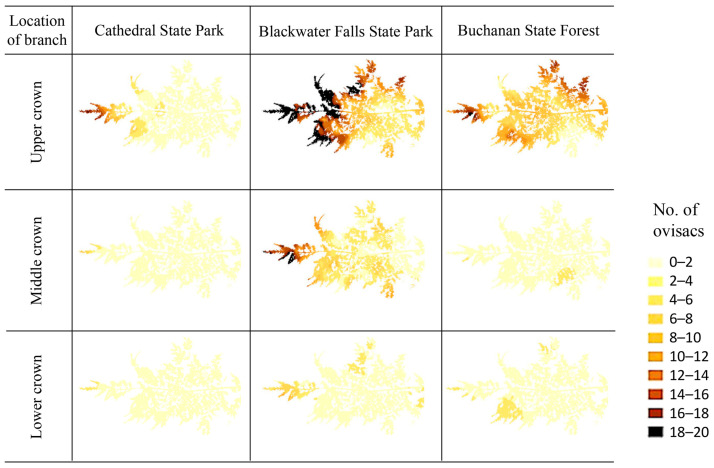
Example within-tree distribution of *A. tsugae* ovisacs at three study sites.

The results of the SADIE showed that approximately 85% of the 744 branches sampled in this study showed a significant (*p* < 0.05) spatial aggregation of *A. tsugae* ovisacs. When the data were pooled by site and vertical location (i.e., upper, middle, and lower crowns), significant spatial aggregations (*I_a_* > 1, *p* < 0.05), except for the middle and lower crowns in Buchanan State Forest, were found (Table 2). More ovisacs were found at the tips of branches except when the ovisac density was very low (Figure 2); the branches with <0.75 ovisacs per 10 cm twig showed random distribution.

#### 3.1.3. Determination of Optimal Sampling Unit

Lower CV values were found mostly in branches sampled from the middle and upper crowns in Cathedral and Blackwater Falls State Parks (i.e., trees < 8 m) and in the lowest branches in Buchanan State Forest (i.e., trees > 15 m) (Table 3). Lower CV values were also found for 50 cm and 100 cm branches in Cathedral and Blackwater Falls State Parks and 50 cm branches in Buchanan State Forest. Considering both short (<8 m) and tall (>15 m) trees, a 50 cm branch is a suitable sampling unit for *A. tsugae* ovisacs. The branches in the middle crown (i.e., ca. 2.9 m above the ground with a range of 2–4 m) among 50 cm branches had the lowest CV values in Cathedral and Blackwater Falls State Parks. In Buchanan State Forest, the branches collected at an average height of 2.8 m from the ground had the lowest CV values. Therefore, based on the CV values, 50 cm branches approximately 3 m above the ground height are suggested as a suitable sampling unit.

#### 3.1.4. Estimating Within-Branch Abundance of *A. tsugae* Ovisacs for the Recommended Sampling Unit

**Figure 3:**
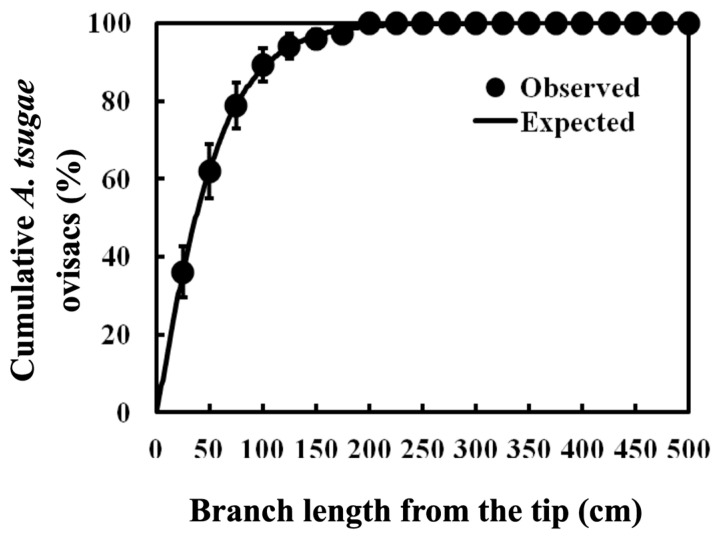
Cumulative ovisac counts (mean ± SE) relative to sample location on the branch 3 m above the ground only when the ovisacs of *A. tsugae* are present. Note that the non-linear regression was modeled with the Weibull function (see text for details).

The cumulative percentage of *A. tsugae* ovisacs on a whole branch ca. 3 m above the ground fit well with the Weibull function (*a* = 50.94 ± 2.964; *b* = 1.14 ± 0.122; *F* = 1801.1; df = 2, 118; *p* < 0.0001; *r*^2^ = 0.97). The selected sampling unit (i.e., 50 cm branch) in this study contained about 71% of the total ovisacs of the branches, while 50% of the ovisacs could be found within 37 cm from the tip of a branch and 99% of ovisacs within 200 cm (Figure 3).

### 3.2. Study 2: Determining Spatial Distribution Patterns of A. tsugae Ovisac

#### 3.2.1. Population Dynamics *A. tsugae* Ovisacs

**Figure 4:**
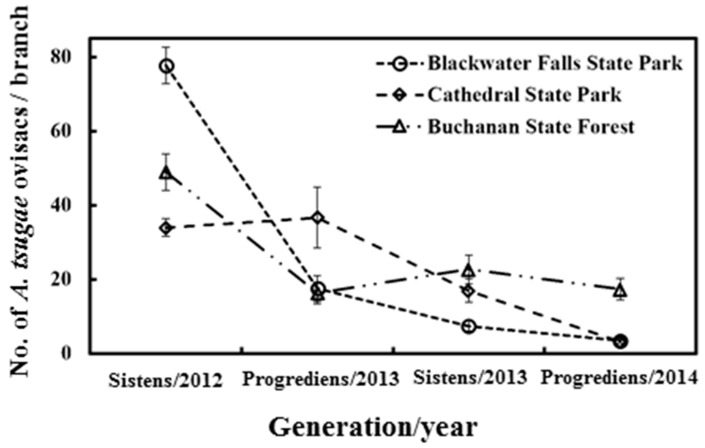
Population dynamics of *A. tsugae* ovisacs. Error bars indicate the standard error of the mean ovisac counts per branch.

The population of *A. tsugae* ovisacs decreased throughout this study (Figure 4). When the data from all three sites were pooled, the population density of *A. tsugae* ovisacs decreased from the first sampling (53.6 ± 2.61 per branch) to the last (8.0 ± 1.04 per branch).

#### 3.2.2. Spatial Distribution Patterns of *A. tsugae*

The results of the semivariogram modeling showed that 10 out of 12 spatial data sets (i.e., three sites × four *A. tsugae* generations) in this study exhibited an apparent spatial structure of *A. tsugae* ovisacs (Table 4). Two cases of semivariograms (progrediens generations of Cathedral State Park in 2013 and Buchanan State Forest in 2014) showed a nugget effect, indicating a random spatial pattern. A total of nine cases of semivariograms fit with a linear model, with slopes suggesting the existence of a spatial trend or drift [39,40]. Therefore, the trends were removed, and residuals were used for the semivariogram modeling [41,42,43]. In Cathedral State Park, the data from all the generations and years exhibited a significant (*p* < 0.05) spatial trend. The linear trend surface models fitting with the dispersion of *A. tsugae* were *y* = 45.58 + 6.151*x*_1_ − 1.108*x*_2_ for the sistens generation in 2012, *y* = –7.930 + 3.047*x*_1_ + 4.564*x*_2_ for the sistens generation in 2013, and *y* = 2.407 + 0.9418*x*_1_ − 0.2267*x*_2_ for the progrediens generation in 2012, where *y* is a function for the trend surface, *x_1_* is the east-west coordinate, and *x_2_* is the north-south coordinate. In Blackwater Falls State Park, all data sets showed a spatial trend: *y* = 107.8 + 0.5424*x*_1_ − 8.140*x*_2_ for the sistens generation in 2012, *y* = 45.26 − 5.935*x*_1_ − 4.051*x*_2_ for the progrediens generation in 2013, *y* = 5.550 − 1.265*x*_1_ +2.941*x*_2_ for the sistens generation in 2013, and *y* = –10.22 − 1.969*x*_1_ + 1.142*x*_2_ for the progrediens generation in 2014. For Buchannan State Forest, spatial trends were detected in three data sets: *y* = 30.44 − 7.11*x*_1_ − 44.31*x*_2_ for the sistens generation in 2012, *y* = 84.83 − 3.19*x*_1_ − 6.28*x*_2_ for the progrediens generation in 2013, and *y* = 129.7 − 4.566*x*_1_ − 12.13*x*_2_ for the sistens generation in 2014. After removing the trends, except the sistens generation of 2012 in Buchannan State Forest, a nugget model fit all the semivariograms best (Table 4), indicating an absence of spatial dependence within the range of *A. tsugae* density observed in this study.

**Figure 5:**
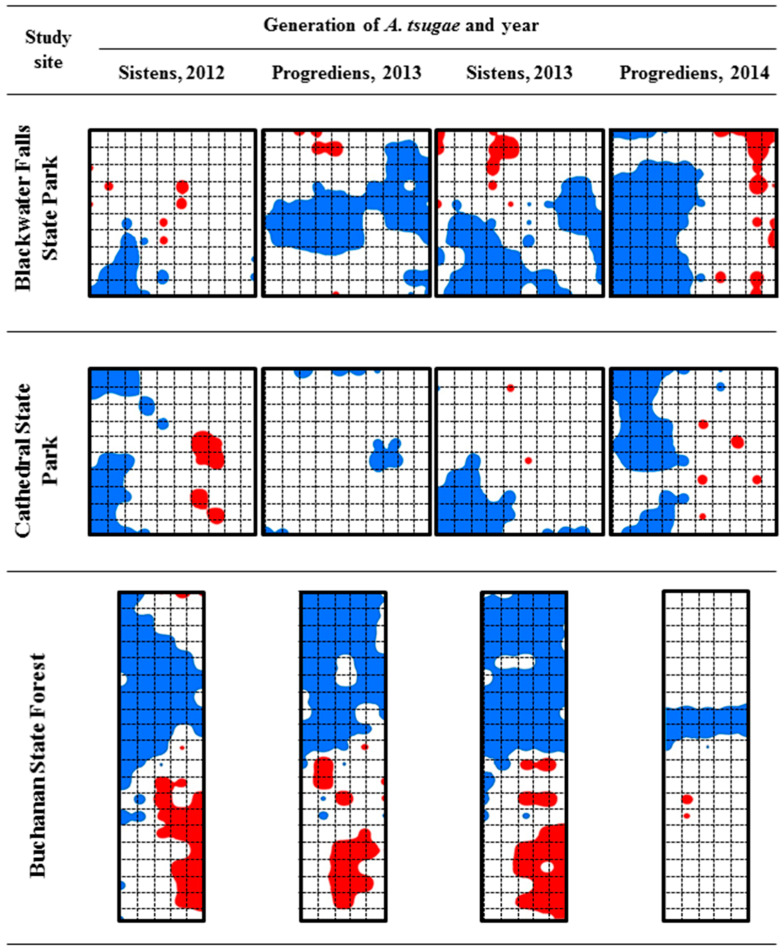
Spatial distribution maps with patches and gaps of *A. tsugae* ovisacs over four generations in three study sites. Red areas indicate patches with clusters of large counts (i.e., *v_i_* > 1.5) and blue areas indicate gaps with clusters of relatively small counts (i.e., *v_i_* < −1.5). The sizes of each site and sample grid are 1 ha and 10 m × 10 m, respectively.

The sistens generation of *A. tsugae* of Buchanan State Forest in 2012 exhibited spatial dependence and fit with the spherical model after removing the trends. Approximately 87% of the spatial variability was explained by spatial dependence, with a range value of 41.3 m (Figure 5).

The results from the SADIE showed that the spatial distribution of *A. tsugae* ovisacs mostly showed spatial aggregation, except for the progrediens generations in Cathedral State Park in 2013 and Buchanan State Forest in 2014, which showed random distribution (Table 5, Figure 5). When all four data sets (four samplings) were pooled by the study site, significant (*p* < 0.05) spatial aggregations of *A. tsugae* ovisacs were found (Table 5).

#### 3.2.3. Spatial Associations between Generations of *A. tsugae*

The spatial associations between the generations of *A. tsugae* ovisacs showed 33% of significant (*p* < 0.025) positive spatial association and 67% of no spatial association (Table 6). Significant and positive spatial association was found between the sistens generation of 2012 and the progrediens generation of 2013, between the progrediens and sistens generations of 2013 in Buchanan State Forest, and between the progrediens and sistens generations of 2013 in Blackwater Falls State Park (Table 6). However, the *A. tsugae* distributions in Cathedral State Park did not show significant spatial associations between generations.

#### 3.2.4. Spatial Association of *A. tsugae* Ovisacs with Environmental Factors

The spatial associations of *A. tsugae* with the surrounding physical and biological environments varied with the study sites. The tree height and DBH showed 33% positive spatial associations with *A. tsugae* distribution (Table 7), mostly in Cathedral State Park. The spatial relationship between *A. tsugae* and the plant species diversity was not consistent across the study sites: 25% positive associations, 25% negative associations, and 50% no relationship (Table 4). Both the elevation and basal area showed positive and significant (*p* < 0.025) associations with *A. tsugae* only in the sistens generation of 2012 in Blackwater Falls State Park and a negative (*p* > 0.975) spatial association in the progrediens generation of 2014 in Blackwater Falls State Park; the other cases showed no spatial relationships. Although the aspect showed 50% positive and significant (*p* < 0.025) spatial relationships with *A. tsugae*, the progrediens generation of 2013 in Blackwater Falls State Park was negatively associated with the aspect.

The number of *Sasajiscymnus tsugae* (Coleoptera: Coccinellidae), a natural enemy of *A. tsugae*, was very low in the three study sites. From the 2400 branches sampled during the 3-year study, only one *S. tsugae* was found in Buchanan State Forest.

## 4. Discussion

### 4.1. Study 1: Determining Optimal Sampling Unit for A. tsugae Ovisac Sampling

Our study indicates that 50 cm branch samples provide a more precise estimation of *A. tsugae* density than 100 cm branch samples. Based on the obtained CV values, we recommend sampling ovisacs from 50 cm branches at approximately 3 m above the ground. The use of 50 cm branches allows for the more efficient detection and counting of *A. tsugae* ovisacs compared to the 100 cm branches, thereby reducing the time and effort spent in the field. To justify the increased effort of counting 50 cm branch segments instead of shorter lengths such as 25 or 30 cm, it is important to consider the trade-offs between sampling effort and data precision. By sampling 50 cm branch sections, the total number of branches required to estimate the *A. tsugae* density with high precision may be reduced, potentially balancing the fieldwork load while improving the accuracy of infestation estimates. Our results showed that sampling 50 cm branches, as opposed to 100 cm branches, led to a reduction in the number of habitable areas and ovisacs by approximately 32% and 29%, respectively, suggesting that smaller, well-targeted samples can yield reliable density estimates with fewer resources.

While shorter branch sections, such as 25 cm sections, could further decrease the time needed for searching and counting ovisacs, this reduction in sampling length may compromise the data quality. New shoots of *T. canadensis* often exceed 25 cm [44], and *A. tsugae* ovisacs are commonly located just after new shoot growth due to the sessile nature of the adelgid after settlement. Therefore, a 25 cm branch sample may not adequately capture the spatial distribution of ovisacs. Consequently, while 25 cm samples could reduce the time in the field, they may also decrease the accuracy and precision of *A. tsugae* density estimates, rendering this approach less effective compared to the use of 50 cm samples.

Our results agreed with those of Evans and Gregoire [23], who observed that the *A. tsugae* density was higher in the upper crown when trees had a low *A. tsugae* population. However, they also reported that such a relationship was reversed when the *A. tsugae* population was high, which was not found in our study. Such variations in the vertical distribution of *A. tsugae* ovisacs might be caused by the small sampling unit size they used, i.e., 30 cm branches. Our results (Figure 3 and Table 3) suggest that sampling 30 cm branches as a sampling unit would sometimes show high CV values (i.e., high variation in sampled values). Although the number and density of *A. tsugae* ovisacs did not show significant differences in all cardinal directions in our study, Evans and Gregoire [19] reported that the *A. tsugae* density was higher on the north-facing branches, probably due to the increased predation or desiccation of *A. tsugae* crawlers on the south-facing branches.

When our suggested sampling unit (i.e., 50 cm branch 3 m above the ground) was compared with that of Costa and Onken [27] (i.e., 100 cm branch), our suggested sampling unit had equal or lower CV values, indicating a lower variability of sampled values (Table 8).

A sistens generation adult produces a single ovisac from March to May while a progrediens adult produces in June and July [45,46]. The ovisacs of *A. tsugae* are commonly used as the first sign of infestation on the most recent shoot growth [20]. Our study showed the spatial aggregation of *A. tsugae* on the tips of branches, except for the middle and lower branches, when the density was very low (e.g., 0.75 ovisacs per 10 cm branch). This pattern was consistent with the findings of Joseph et al. [21], who reported that the ovisac density was significantly higher on the tips of branches in the lower and middle crowns but not in the upper crown. This pattern is associated with the distribution of the new shoot growth of hemlock because *A. tsugae* settles at the bases of needles on new growth on branches and their movement is limited [47]. The reason for nymphal settlement at the bases of needles on new growth is that *A. tsugae* can maximize their access to the vascular connections with adjacent leaves [48] and the quantity of monoterpenes from new shoots is different than that from the previous year’s shoots [49]. However, *A. tsugae* can also be established on older twigs as its population increases [45]. Such a within-branch distribution of *A. tsugae* ovisacs might lead to using branch tips as a sampling unit for *A. tsugae*.

Joseph et al. [21] tried to estimate the population density of *A. tsugae* ovisacs on a branch using 10 cm and 30 cm branch tips from 60 cm branches. They used linear regression and found that 75% and 95% of the variation in the data was explained with the regressions of 10 cm and 30 cm branch tips, respectively. However, this method may not be used to estimate the total number of *A. tsugae* ovisacs on whole branches because they developed their model based on 60 cm branches. In our study, we used a whole branch and a non-linear regression (i.e., Weibull function), and a 97% variation in the data could be explained in estimating the cumulative percentage of *A. tsugae* ovisacs within a branch. This result could be used to estimate the total ovisac number within a branch by counting the ovisacs in a part of the branch and to compare results among studies using different sampling units.

### 4.2. Study 2: Determining Optimal Sampling Unit for A. tsugae Ovisac Sampling

The spatial distributions of insect populations commonly show some degree of spatial aggregation because the available resources and environmental factors are not uniform [50]. In this study, we found that the *A. tsugae* population mostly (10 of 12 samplings) showed spatial trends according to the semivariograms and spatial aggregation according to the SADIE results, regardless of the study site and *A. tsugae* generation. The spatial trends and aggregation of *A. tsugae* can be caused by heterogeneities of local environmental conditions and its limited ability to move between trees. Because mobile *A. tsugae* crawlers settle at the base of needles on new twig growth [47], the *A. tsugae* population may disperse slowly from the infested trees. We found a random distribution of the progrediens generation of 2014 in Cathedral State Park, which might be caused by unusual storm damage that caused approximately 50% of trees to fall. The progrediens generation of *A. tsugae* in 2013 from Buchanan State Forest also showed random distribution, which might be caused by the high mortality of *A. tsugae* during winter. Skinner et al. [51] reported that only 14% of the adelgids could survive after exposure to −15 °C, which was the case in Buchanan State Forest which was at a higher latitude compared to other sites.

Our study showed that 67% of the *A. tsugae* ovisacs had no spatial association across generations, while 33% had positive spatial associations. It is known that *A. tsugae* is sessile except for the crawler stage and passively disperses with the help of wind, birds, deer, and humans [52]. Limited movement or dispersal abilities might explain the positive spatial association between two consecutive sampling periods. However, the spatial association may be dependent upon the *A. tsugae* density. McClure [14] reported that *A. tsugae* populations showed peaks every two years. In our study, new shoot development of eastern hemlocks was limited during the first year of the infestation by *A. tsugae.* As a result, *A. tsugae* populations might decrease in the second year. In the third year, the *A. tsugae* population might increase a little bit due to newly developed shoots. This population cycle is determined by the population density of *A. tsugae* [14].

Environmental factors also affect the spatial distribution patterns of *A. tsugae.* We found that the basal area and elevation showed spatial association with the *A. tsugae* ovisacs in most cases. This result agreed with the findings by Orwig et al. [25], who showed a significant association between elevation and *A. tsugae* populations in a non-spatial study. Our study also showed that the spatial associations of the DBH, tree height, and aspect with *A. tsugae* ovisacs showed some significant spatial associations, although their spatial association was site-specific and *A. tsugae*-generation specific. These results were consistent with previous studies done without consideration of the spatial distribution. Orwig et al. [25] reported that the aspect was significantly correlated with the *A. tsugae* population and Rentch et al. [26] found that the aspect and overtopped trees were correlated with *A. tsugae*. They also found that the occurrence of *A. tsugae* populations was not related to the decline of eastern hemlock due to the compensation of trees. Codominant trees and east- or south-facing trees receive more light and then maximize new shoot growth [26]. The increased number of new shoots might accommodate more *A. tsugae* populations.

## 5. Conclusions

The results of our studies imply three important considerations in *A. tsugae* sampling and management. First, currently, foresters and park managers seek information on where to prioritize *A. tsugae* management because all hemlock stands cannot be treated with chemicals, partially due to the high cost of chemical control. In this study, we found the spatial aggregation of *A. tsugae* by SADIE and spatial trends by semivariograms in most cases, which can be used for planning site-specific management for *A. tsugae.* Site-specific insect pest management is a strategy based on local insect densities within an area rather than the uniform management of insect pests based on average densities throughout the area [53]. In addition, because *T. canadensis* is distributed along streams [3] and located in broad areas, the site-specific pest management strategy may provide a solution to reduce pesticide input and maximize the control by pesticide [54] by detecting areas of *A. tsugae* aggregation and applying control measures in the selected areas. Second, the results of semivariogram modeling can provide the distance of sample locations to obtain independent samples. Sampling plans for *A. tsugae* have been developed [22,27], but no suggestions have been made on how far apart two adjacent samples need to be to obtain independent samples. In our study, a semivariogram showed a nugget model in 11 out of 12 sample dates after removing the spatial trends, indicating any distance > 10 m (i.e., sample distance of this study) can be independent. However, the semivariogram of the sistens generation of 2012 in Buchannan State Forest fit with a spherical model with a range value of 41.3 m, indicating that any two samples are independent when they are at least 41.3 m apart. This distance can be used as a conservative minimum distance between two samples for currently available sampling plans (i.e., binomial and sequential sampling plans). Third, we found that *A. tsugae* ovisacs were spatially associated with some environmental factors depending on the sites. In our study, the DBH, tree height, and aspect in the Cathedral State Park site were key factors determining *A. tsugae* distribution. Mapping *A. tsugae* populations is not an easy task and, thus, these factors can be mapped economically and used to describe *A. tsugae* distribution. Furthermore, these environmental factors could be used as covariates for co-kriging to generate an *A. tsugae* distribution map; co-kriging is a geostatistical procedure to generate distribution maps that area specifically used when intensive sampling of a main attribute (i.e., *A. tsugae*) is limited while intensive sampling for the related variables (i.e., DBH, tree height, and aspect) is achievable [34].

## Figures and Tables

**Table 1:** Results of ANOVA for the number and density of *A. tsugae* ovisacs per 1 cm twig with foliage in four different cardinal directions (east, west, south, and north) and six different branch heights for sampling.

Sites and Sample Variables	Length of Sampled Branch					
	100-cm Branch			Whole Branch		
	*F*	*df*	*p*	*F*	*df*	*p*
Cathedral State Park						
Cardinal direction						
No. of ovisacs per branch	1.51	3, 236	0.22	0.08	3, 236	0.97
Density of ovisacs per branch	1.06	3, 236	0.37	0.06	3, 236	0.98
Branch height						
No. of ovisacs per branch	6.95	5, 234	<0.01	1.35	5, 234	0.25
Density of ovisacs per branch	6.60	5, 234	<0.01	2.84	5, 234	0.02
Blackwater Falls State Park						
Cardinal direction						
No. of ovisacs per branch	0.32	3, 620	0.81	0.02	3, 620	0.99
Density of ovisacs per branch	0.14	3, 620	0.94	0.13	3, 620	0.94
Branch height						
No. of ovisacs per branch	17.01	5, 618	<0.01	24.71	5, 618	<0.01
Density of ovisacs per branch	14.10	5, 618	<0.01	41.67	5, 618	<0.01
Buchanan State Forest						
Cardinal direction						
No. of ovisacs per branch	0.61	3, 236	0.61	1.39	3, 236	0.25
Density of ovisacs per branch	0.94	3, 236	0.42	0.61	3, 236	0.61
Branch height						
No. of ovisacs per branch	2.84	5, 234	0.02	1.65	5, 234	0.15
Density of ovisacs per branch	2.94	5, 234	0.02	13.33	5, 234	<0.01

**Table 2:** Spatial distribution patterns of *A. tsugae* ovisacs measured with SADIE index, *I*_a_. The overall degree of clustering (*I*_a_) with its associated *p*-value (*p*_a_) is shown in parentheses. *I*_a_ = 1 suggests a random, *I*_a_ > 1 suggests an aggregated, and *I*_a_ < 1 suggests a regular spatial pattern. Significant (*p* < 0.05) associations are in bold.

Location of Sampled Branch	Study Site		
	Cathedral State Park	Blackwater Falls State Park	Buchanan State Forest
Upper crown	**3.27 (0.01)**	**7.05 (0.01)**	**1.35 (0.03)**
Middle crown	**4.11 (0.01)**	**10.88 (0.01)**	1.22 (0.13)
Lower crown	**1.92 (0.01)**	**2.02(0.01)**	1.40 (0.06)

**Table 3:** Coefficients of variance (CVs) for the number of *A. tsugae* ovisacs in six different vertical locations for sampling.

Site	Crown Location	Branch Length ^a^		
		25 cm	50 cm	100 cm
Cathedral State Park	Lower crown, bottom half	1.82	1.75	2.03
	Lower crown, top half	1.17	1.64	2.36
	Middle crown, bottom half	1.49	1.57	1.62
	Middle crown, top half	1.10	1.14	1.11
	Upper crown, bottom half	1.00	1.17	1.12
	Upper crown, top half	1.12	0.95	0.93
Blackwater Falls State Park	Lower crown, bottom half	1.47	1.30	1.22
	Lower crown, top half	1.24	1.24	1.16
	Middle crown, bottom half	1.48	1.32	1.21
	Middle crown, top half	1.24	1.17	1.22
	Upper crown, bottom half	1.21	1.23	1.23
	Upper crown, top half	1.29	1.37	1.38
Buchanan State Forest	Lower crown, bottom half	1.28	1.01	1.03
	Lower crown, top half	1.77	1.34	1.27
	Middle crown, bottom half	2.01	1.78	1.51
	Middle crown, top half	1.40	1.49	1.44
	Upper crown, bottom half	1.22	1.09	1.24
	Upper crown, top half	1.35	1.16	1.34

^a^ Length of a branch sample cut from the tip of the branch.

**Table 4:** Semivariogram parameters to characterize spatial distribution patterns of *A. tsugae* ovisacs.

Site	*A. tsugae* Generation and Year	Density ^a^	Model	Nugget	Sill	Range (m)	r^2^
Blackwater Falls State Park	Sistens, 2012 ^b^	156.8	Nugget	9257	9257	N/A ^c^	<0.01
	Progrediens, 2013 ^b^	35.2	Nugget	3681	3681	N/A	<0.01
	Sistens, 2013 ^b^	14.8	Nugget	638.6	638.6	N/A	<0.01
	Progrediens, 2014 ^b^	7.0	Nugget	42.3	42.3	N/A	<0.01
Cathedral State Park	Sistens, 2012 ^b^	68.0	Nugget	26431	26,431	N/A	<0.01
	Progrediens, 2013	74.0	Nugget	1.930	1.930	N/A	<0.01
	Sistens, 2013 ^b^	34.2	Nugget	3982	3982	N/A	<0.01
	Progrediens, 2014 ^b^	6.4	Nugget	81.93	81.93	N/A	<0.01
Buchannan State Forest	Sistens, 2012 ^b^	98.7	Spherical	4500	19,680	41.3	0.897
	Progrediens, 2013 ^b^	32.8	Nugget	2848	2848	N/A	<0.01
	Sistens, 2013 ^b^	45.3	Nugget	4601	4601	N/A	<0.01
	Progrediens, 2014	34.7	Nugget	3824.7	3827.7	N/A	<0.01

^a^ Number of *A. tsugae* ovisacs per branch. ^b^ Spatial trends were removed with trend surface analysis before semivariogram modeling. ^c^ Not applicable.

**Table 5:** Spatial distribution patterns of *A. tsugae* ovisacs measured with SADIE index, *I*_a_. The overall degree of clustering (*I*_a_) with its associated *p* value (*p*_a_) is shown in parentheses. *I*_a_ = 1 suggests a random, *I*_a_ > 1 suggests an aggregated, and *I*_a_ < 1 suggests a regular spatial pattern. Significant (*p* < 0.05) associations are in bold.

Study Site	*A. tsugae* Generation and Year				
	Sistens, 2012	Progrediens, 2013	Sistens, 2013	Progrediens, 2014	Pooled
Blackwater Falls State Park	**1.35 (0.03)**	**1.49 (0.01)**	**1.73 (0.01)**	**1.91 (0.01)**	**1.56 (0.01)**
Cathedral State Park	**1.55 (0.01)**	0.99 (0.46)	**1.32 (0.04)**	**1.61 (0.01)**	**1.35 (0.03)**
Buchanan State Forest	**2.59 (0.01)**	**2.33 (0.01)**	**2.61 (0.01)**	0.95 (0.46)	**2.97 (0.01)**

**Table 6:** Spatial association between two generations of *A. tsugae* based on ovisac counts. The index of association (*X*) with its associated *p* value (*p*_t_) is shown in parentheses. For a two-tail test at a 95% confidence level, *p*_t_ < 0.025 indicates a significant positive association and *p*_t_ > 0.975 indicates a significant negative association. Significant associations are in bold.

Study Site	*A. tsugae* Generations Compared		
	Sistens, 2012 vs. Progrediens, 2013	Sistens, 2013 vs. Progrediens, 2013	Sistens, 2013 vs. Progrediens, 2014
Blackwater Falls State Park	−0.09 (0.79)	**0.36 (< 0.01)**	−0.02 (0.59)
Cathedral State Park	0.06 (0.28)	0.12 (0.13)	0.13 (0.11)
Buchanan State Forest	**0.36 (<0.01)**	**0.49 (<0.01)**	0.18 (0.04)

**Table 7:** Spatial associations between *A. tsugae* ovisacs and surrounding environmental factors. Index of association (*X*) with its associated *p* value (*p*_t_) is shown in parentheses. For a two-tail test at a 95% confidence level, *p*_t_ < 0.025 indicates a significant positive association and *p*_t_ > 0.975 indicates a significant negative association.

Study Site	*A. tsugae* Generation and Year	DBH ^a^	Tree Height	Basal Area	Diversity ^b^	Elevation	Aspect
Blackwater Falls State Park	Sistens, 2012	−0.03 (0.61)	0.05 (0.30)	0.02 (0.41)	0.09 (0.19)	0.28 (<0.01)	0.14 (0.09)
	Progrediens, 2013	−0.11 (0.85)	0.22 (0.02)	0.19 (0.03)	0.18 (0.04)	−0.04 (0.66)	−0.28 (0.99)
	Sistens, 2013	−0.18 (0.96)	0.12 (0.12)	0.01 (0.47)	0.32 (<0.01)	−0.03 (0.62)	−0.08 (0.74)
	Progrediens, 2014	−0.01 (0.52)	−0.02 (0.56)	−0.25 (0.99)	−0.24 (0.99)	0.11 (0.14)	0.72 (<0.01)
Cathedral State Park	Sistens, 2012	0.29 (<0.01)	0.25 (<0.01)	−0.07 (0.77)	−0.37 (1.00)	0.18 (0.04)	0.46 (<0.01)
	Progrediens, 2013	−0.07 (0.76)	0.01 (0.47)	0.08 (0.21)	−0.03 (0.61)	0.12 (0.13)	0.29 (0.01)
	Sistens, 2013	0.30 (<0.01)	0.34 (<0.01)	0.12 (0.13)	−0.27 (0.99)	0.07 (0.24)	0.18 (0.05)
	Progrediens, 2014	0.35 (<0.01)	0.31 (<0.01)	0.05 (0.68)	−0.05 (0.67)	0.05 (0.31)	0.37 (<0.01)
Buchanan State Forest	Sistens, 2012	0.28 (<0.01)	0.21 (0.01)	−0.11 (0.85)	0.21 (0.02)	-	-
	Progrediens, 2013	0.09 (0.17)	0.08 (0.20)	0.09 (0.17)	0.35 (< 0.01)	-	-
	Sistens, 2013	0.20 (0.04)	0.18 (0.05)	0.20 (0.04)	0.09 (0.19)	-	-
	Progrediens, 2014	−0.03 (0.63)	−0.10 (0.84)	−0.18 (0.93)	−0.03 (0.61)	-	-

^a^ Diameter at breast height; ^b^ Simpson’s diversity index.

**Table 8:** Comparison of coefficients of variance (CVs) between the current sampling unit (i.e., the lowest 100 cm branches; Costa and Onken 2009) and the suggested sampling unit from this study (i.e., 50 cm branches 3 m above the ground).

Site	Sampling Unit	CV Value
Cathedral State Park	Current	1.85
	As suggested by this study	1.14
Blackwater Falls State Park	Current	1.17
	As suggested by this study	1.17
Buchanan State Forest	Current	1.03
	As suggested by this study	1.01

## Data Availability

Data are contained within the article.

## Supplementary Materials

The following supporting information can be downloaded at: https://www.mdpi.com/article/10.3390/insects15100751/s1, Figure S1: Semivariogram depicting the spatial structure of *A. tsugae* distribution found in the site of Buchanan State Forest in 2012. Note that spatial dependence was detected up to 41.7 m (i.e., range value) as indicated by the vertical dash line; Table S1: *A. tsugae* ovisac density measured from whole-branch samples. The ovisac density was presented in two ways: the number of ovisacs per branch and the number of ovisacs per habitable space in the branch; Table S2: *A. tsugae* ovisac density measured from 100-cm-branch samples. The ovisac density was presented in two ways: the number of ovisacs per branch and the number of ovisacs per habitable space in the branch.
