# Supplementary material for: Spatial Patterns of Adelges tsugae Annand (Hemiptera: Adelgidae) in Eastern Hemlock Stands: Implications for Sampling and Management
Source: Insects. 2024 Sep 28;15(10):751. doi: 10.3390/insects15100751 (PMC11508703; doi:10.3390/insects15100751)
Supplement: Supplementary file 1 [file insects-15-00751-s001.zip › insects-3167245-supplementary.pdf]

Table S1. *Adelgis tsugae* ovisac density measured from whole-branch samples. The ovisac density was presented in two ways: the number of ovisacs per branch and the number of ovisacs per habitable space in the branch

Branch location	Cathedral State Park		Blackwater Falls State Park		Buchanan State Forest	
	No. / branch	No. / habitable space	No. / branch	No. / habitable space	No. / branch	No. / habitable space
Lower crown, bottom half	88 ± 36.6a [a]	0.5 ± 0.21b	6 ± 2.1c	0.1 ± 0.03c	656 ± 111.2a	2.5 ± 0.34c
Lower crown, top half	167 ± 76.8a	0.7 ± 0.21ab	4 ± 1.6c	0.1 ± 0.03c	966 ± 159.6a	2.6 ± 0.37c
Middle crown, bottom half	297 ± 86.7a	1.1 ± 0.27ab	37 ± 16.8bc	0.2 ± 0.06c	993 ± 162.3a	3.9 ± 0.73c
Middle crown, top half	200 ± 52.0a	1.4 ± 0.38ab	86 ± 21.8abc	0.7 ± 0.11bc	847 ± 142.7a	5.2 ± 0.88bc
Upper crown, bottom half	179 ± 48.1a	1.9 ± 0.48ab	177 ± 48.5a	1.5 ± 0.29b	1,075 ± 184.9a	7.2 ± 0.77ab
Upper crown, top half	104 ± 33.9a	2.2 ± 0.58a	108 ± 23.9ab	3.3 ± 0.66a	662 ± 135.1a	9.3 ± 0.96a

[a] Means followed by the same letter within a column are not significantly different at $\alpha = 0.05$, Tukey HDS.

Table S2. *Adelgis tsugae* ovisac density measured from 100-cm-branch samples. The ovisac density was presented in two ways: the number of ovisacs per branch and the number of ovisacs per habitable space in the branch

Branch location	Cathedral State Park		Blackwater Falls State Park		Buchanan State Forest	
	No. / branch	No. / habitable space	No. / branch	No. / habitable space	No. / branch	No. / habitable space
Lower crown, bottom half	32 ± 7.2c [b]	0.6 ± 0.10b	145 ± 16.7c	3.0 ± 0.47d	106 ± 26.9ab	2.2 ± 0.69ab
Lower crown, top half	42 ± 7.4c	0.5 ± 0.07b	282 ± 29.4c	3.9 ± 0.41d	53 ± 18.8b	0.6 ± 0.20ab
Middle crown, bottom half	71 ± 13.5c	0.5 ± 0.07b	397 ± 44.6abc	5.7 ± 0.67cd	22 ± 6.8b	0.3 ± 0.14b
Middle crown, top half	117 ± 15.5bc	0.8 ± 0.09b	630 ± 73.2ab	8.5 ± 0.99bcd	13 ± 6.0b	0.3 ± 0.24b
Upper crown, bottom half	268 ± 61.8a	2.3 ± 0.63b	701 ± 84.7a	10.2 ± 1.06a	201 ± 100.2ab	2.3 ± 1.42ab
Upper crown, top half	237 ± 29.6ab	4.7 ± 1.15a	648 ± 88.4a	12.4 ± 1.45ab	401 ± 172.6a	3.8 ± 1.40a

[a] Means followed by the same letter within a column are not significantly different at $\alpha = 0.05$, Tukey HDS.

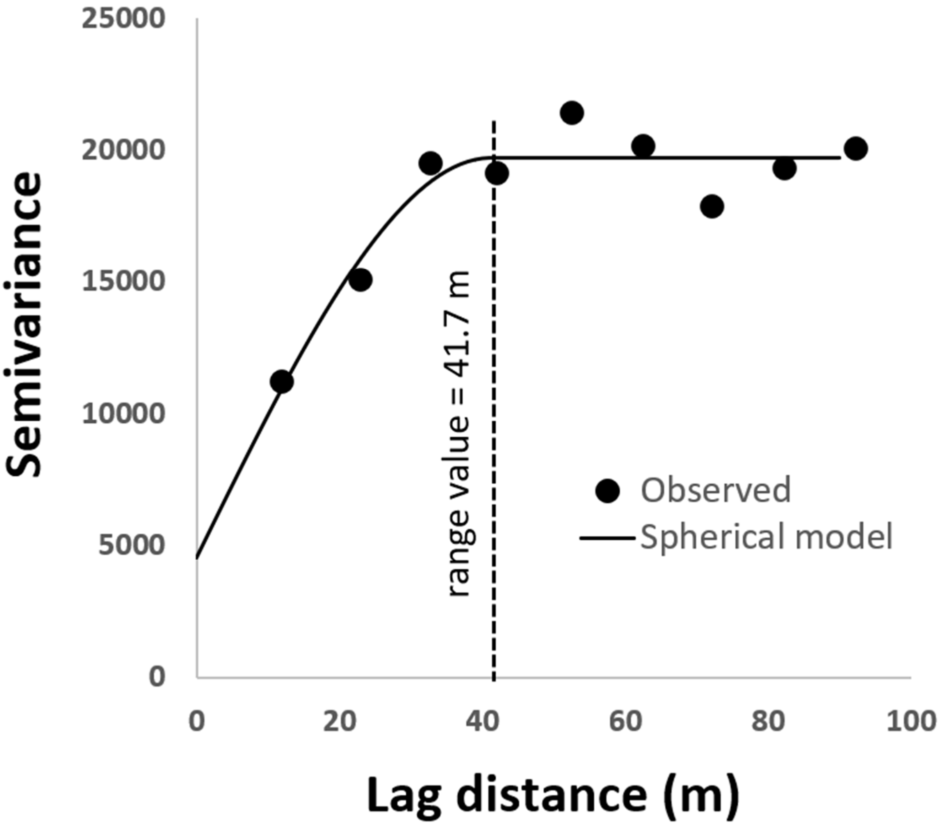

Fig. S1. Semivariogram depicting the spatial structure of *A. tsugae* distribution found at the site of Buchanan State Forest in 2012. Note that spatial dependence was detected up to 41.7 m (i.e., range value) as indicated by the vertical dash line.
